# The influence of *Nrf2* gene promoter methylation on gene expression and oxidative stress parameters in preeclampsia

**DOI:** 10.1186/s12920-023-01791-6

**Published:** 2024-02-28

**Authors:** Saba Zakeri, Zohreh Rahimi, Nayebali Rezvani, Asad Vaisi-Raygani, Reza Alibakhshi, Sahel Zakeri, Kheirolah Yari

**Affiliations:** 1 Department of Clinical Biochemistry, Medical School, Kermanshah University of Medical Sciences, Daneshgah Avenue, Kermanshah, P.O.Box: 67148-69914, Iran; 2 Medical Biology Research Center, Health Technology Institute, Kermanshah University of Medical Sciences, Kermanshah, Iran; 3 Department of Biology, Faculty of Basic Sciences, Islamic Azad University, Sanandaj, Iran

**Keywords:** Preeclampsia, Epigenetic, *Nrf2* gene, Placenta, Oxidative stress

## Abstract

**Background and aims:**

Preeclampsia (PE) is a serious medical condition that usually causes high blood pressure and affects multiple organs. Considering the adverse effect of oxidative stress on the process of PE in pregnant women and regarding the role of the *Nrf2* gene in placental oxidative pathways, this study was conducted to investigate the DNA methylation status of *Nrf2* in PE and healthy pregnant women.

**Materials and methods:**

The present case-control study consisted of 70 PE and 70 healthy pregnant women. Blood and placenta samples were taken from all subjects, and the percentage of the *Nrf2* gene methylation in the samples was assessed by the Methyl Light PCR method. Also, the *Nrf2* gene expression was evaluated by real-time PCR. The total antioxidant capacity (TAC) and total oxidative status (TOS) were measured by the colorimetric method.

**Results:**

In PE women, there was a significant increase in blood pressure, term of pregnancy, and BMI. In addition, there were enhanced *Nrf2* DNA methylation percentage in placenta tissue and increased TOS levels in placenta tissue and blood compared to healthy pregnant women (*P* < 0.05). Also, in the PE group, there was a significant decrease in *Nrf2* gene expression and TAC level in placenta tissue compared to the control group (*P* < 0.05).

**Conclusion:**

The *Nrf2* gene undergoes epigenetic modifications of DNA hypermethylation in the PE placenta. Decreased expression of this gene and the changes in the level of oxidative parameters (TAC, TOS) confirm it.

## Introduction

Preeclampsia (PE) is a crucial medical condition that usually occurs after 20 weeks of gestation; it commonly induces high blood pressure and affects some organs, including the liver, kidney, and brain. So, it accounts for a threatening crisis for the mother and the fetus [1]. This condition is characterized by placenta defects, dysfunction in angiogenesis such as abnormal regeneration of spiral artery, high level of anti-angiogenic factors and low level of pro-angiogenic factors, oxidative stress at the maternal-fetal interface, and angiogenic imbalance in maternal blood circulation [2]. It is reported that PE appears in 3–5% of pregnancies and is the cause of 42,000 deaths per year [3]. In addition, PE causes morbidity (acute and long-term), perinatal deaths, stillbirths, and intrauterine growth restriction [4]. In the process of pregnancy, the remodeling of the uterine spiral arteriole is necessary for the exchange of food and oxygen between the mother and the fetus, and its disruption causes insufficient blood supply to the placenta, high blood flow speed, and disturbed blood flow. The outcome of these blood circulation disorders is placental ischemia, oxidative stress, and placental villi damage [5, 6]. On the other hand, the consequences that affect the mother’s health include high levels of angiogenic protein, vascular inflammation, endothelial dysfunction, vascular damage, and clinical manifestations such as hypertension and damage to several organs in the mother [7].

In previous research, studies have been done on the role of genetic factors related to histo-compatibility, vascular disorders, metabolic changes, inflammatory mediators, detoxification, DNA repair, and apoptosis in PE [8]. In addition to genetic factors, epigenetic events play a role during normal and pathological pregnancy. These factors create, maintain, and regulate chromatin structure via DNA methylation, post-translational histone modifications, and regulatory non-coding RNAs. This suggests that the mechanisms that cause phenotypic changes during pregnancy and at birth are, at least in part, epigenetic [9]. In this context, studies have done on labor-related inflammatory genes (*PTGS2, BMP2, NAMPT/PBEF, CXCL2*), steroid receptor genes (*ESR1, PGR, NR3C1/GR*) and components of the renin-angiotensin system (*ACE, ATP6AP2/PRR, AGTR1, CTSD, KLK1*) with promoter methylation density technique in amnion samples from early pregnancy (11 to 17 weeks) and after delivery [9].

Previous studies revealed that the nuclear factor erythroid-2-related factor-2 (*Nrf2*) gene is known to protect cells against redox-mediated damage and carcinogens. *Nrf2* activity is regulated by Kelch-like ECH-associated protein 1 (Keap1), a redox-sensitive E3 ubiquitin ligase substrate adaptor; so that, under homeostatic conditions, two molecules of Keap1 are bound to the *Nrf*_*2*_ and cause ubiquitination and degradation of the *Nrf*_*2*_ [10]. In response to oxidative stress, Keap1 is oxidized and inactivated, *Nrf*_*2*_ stabilizes and translocates into the nucleus, and heterodimerizes with the small musculoaponeurotic fibrosarcoma (sMaf) protein family (MafF, MafG, and MafK) [11]. The Nrf_2_-sMaf complex binds to the antioxidant response element (ARE 5-TGACXXXGC-3) and induces genes to protect the cell against oxidative stress. The mutations of Nrf2 result in the modification of the Nrf2 protein residues that interact with Keap1 causing activation of the cap’n’collar (CNC)– basic leucine zipper protein (bZIP) transcription factor [12] and leading to upregulation of *Nrf2/ARE* gene transcription [13, 14]. Previous studies showed that Nrf_2_/ Keap1 signaling play a key role in the pathology of cancers such as ovarian [15], prostate [16], cervical, and endometrial cancers [17]. In addition, in the study of Lost et al., it was shown that the genes belonging to the Nrf_2_/ Keap1 signaling pathway are expressed more than other genes among the decidu genes of PE patients [18].

Among the characteristics of PE is the increase in lipid oxidation and reduced antioxidant capacity. Vascular Endothelial growth factor (VEGF) affects preeclampsia and prevents oxidative damage by activating the *Nrf2* path. It has been shown that in cases of Intrauterine Growth Restriction/PE, there is an increase in *Nrf2* expression and reduced VEGF expression [19].

Due to the effect of oxidative stress on the process of preeclampsia and given the role of the *Nrf2* gene in oxidative pathways in the placenta, this study was accomplished to detect the methylation states and the gene expression of the *Nrf2* in healthy and preeclampsia pregnant women. In addition, we measured the levels of the total oxidant status (TOS) and total antioxidant capacity (TAC) [20] in the same samples to unveil the effect of the *Nrf2* gene expression fluctuations and its methylation changes on the level of oxidative parameters.

## Materials and methods

### Study design and population

This study was approved by the Ethics Committee of Kermanshah University of Medical Sciences (IR. KUMS. REC.1399.1078) and consent was obtained from the participants.

The population in this case-control study consisted of 140 pregnant women from Western and Northern Iran with definitive diagnosis of preeclampsia based on two criteria: (a) high maternal blood pressure (systolic > 140 mm Hg and diastolic > 90 mm Hg; with two measurements at an interval of 6 h), and (b) proteinuria > 300 mg in the urine sample of PE women. Inclusion criteria in both groups were all women with a history of first singleton pregnancy, who had visited the medical centers under our study to go through the delivery. Exclusion criteria in both groups include a history of chronic hypertension, chronic kidney disease, liver disease, asthma, diabetes, cardiovascular disease, autoimmune disease, history of stillbirth, placental abruption, infertility, artificial pregnancy, age > 40 years, twin or multiple births, the fetus was affected by hydatid form mole and fetal hydrops based on the patient’s medical record.

### The Nrf2 methylation assay

#### DNA extraction from blood and tissue samples

DNA extraction from whole blood samples and placenta tissues was done by the phenol-chloroform method [21]. Red and white blood cell lysis buffers were used before using sodium dodecyl sulfate (SDS) lysis buffer. Proteinase K was used for the enzymatic digestion of proteins and cellular components in whole blood and placental homogenized tissue samples, and phenol-chloroform was used to denature cellular proteins. Ethanol (70%) was used for DNA precipitation. In the last step, the extraction product was dissolved in ultrapure water to check the DNA concentration with the NanoDrop™ spectrophotometer (ND-1000 Spectrophotometer, *Saveen Werner*, Malmö, Sweden).

#### Bisulfite modification and DNA purification

The logic of using the DNA bisulfite method is that non-methylated DNA cytosine is de-aminated in the exposure of sodium bisulfite and turns into uracil, but methylated cytosine does not change [22]. Bisulfite conversion and subsequent purification were performed according to the manufacturer’s instructions (EZ DNA Methylation-Lightning Kit, Zymo Research, California, USA). To do this step, we mixed 130µL Ct conversion reagent with 20µL extracted DNA, span, and according to the following thermocycling program was put in thermocycler set MJ Mini Personal Thermal Cycler): Initial denaturation (98˚C, 5 min); second denaturation (64˚C, 150 min); and keeping at 4˚C. After the treatment of DNA with sodium bisulfite, DNA purification was performed as below;

After adding 600 µL of M-Binding buffer to the Zymo-Spin™ IC column, the DNA samples were added to the column. After mixing, the sample was washed and centrifuged two times with the M-Wash buffer (> 10,000 x g; 30 s).

In the next step, 200µL M-Desulphonation buffer was added to the column; after 5 min of incubation at room temperature, it was washed and centrifuged (> 10,000 x g; 5 s).

After two washing steps, the column was placed in the micro-centrifuge tube, and M-elution buffer (10 µL) was added to the column matrix and centrifuged for 5 s to elute the DNA.

Using the spectrometer *Nano Drop* the purity, quality, and quantity of DNA after bisulfite modification were determined. The DNA quantity was 200–500 ng/µL in blood and tissue specimens by measuring UV absorbance at 260 nm. Protein impurity was measured according to its absorption at the wavelength of 260/280 nm. To assay the contamination, the absorbance of the samples was measured at the wavelength of 230/260.

#### MethyLight PCR method

In this method, a pair of primers (forward and reverse) and a probe were used to detect the methylated sequence of the *Nrf2* gene promoter region in bisulfite DNA. To perform MethyLight PCR, we used the High ROX master mix prepared by Biosystem Company, a pair of primer, and a TaqMan probe designed by Beacon Designer software 8.13. The reference gene in this study, to normalize the PCR reaction DNA, was *ALU*-*C4*; a pair of primers and a probe (lacking CpG dinucleotide) was designed for the reference gene as well.

#### Designing the probe and primers

The sequence of the *Nrf2* gene promoter region was taken from the UCSC Genome database.

The specific primers and probes for the *Nrf2* and *ALU-C4* gene promoters were designed using Beacon Designer™ (version 8.13; www.premierbiosoft.com/molecular beacons; USA).

Designed primers and probes were synthesized by Metabion (Steinkirchen, Germany) (Table 1).

**Table 1:** Primer and probe sequences used for MethyLight polymerase chain reaction

Primers and probes	Sequence
*Nrf2* Forward Primer	5’-GGGGATTTTCGGAGTTTAGTTCGC-3’
*Nrf2* Reverse Primer	5’CGACTACACGCCGACGAACC-3’
*Nrf2* Probe	5’-6-FAM-CGCTCCGCCTCCCGCCCCACAAAA-BHQ-1-3’
*ALU-C4* Forward primer	5’-GGTTAGGTATAGTGGTTTATATTTGTAATTTTAGTA-3’
*ALU-C4* Reverse primer	5’-ATTAACTAAACTAATCTTAAACTCCTAACCTCA-3’
*ALU-C4* Probe	5’-6-FAM-CCTACCTTAACCTCCC-BHQ-1-3’

#### MethyLight quantitative polymerase chain reactions (qPCR)

This step was performed by PCR 7500 Real-Time (Applied Biosystems; Thermo Fisher Scientific, Inc.), and according to the instructions of the PCRBIOSYSTEMS kit containing qPCRBIO Prob-Mix and ROX Dye solution (Lot.PB026621). The total volume of this reaction was 20 µL, which contained 0.8 µL forward primer,0.8 µL reverse primer,0.4 µL probe,3 µL DNA sample,0.2 µL ROX Dye, and 5 µL PCR grade dH2O.

The temperature cycle of this reaction was as follows:

PCR primary activation stage (95˚C, 3 min), denaturation stage (95˚C, 5 s), annealing/extension stage with fluorescence data collection (57^0^C, 30 s).

This reaction was performed in a special strip for ABI 7500 real-time device for Nrf2 and ALU-C4 sequences, with positive control (BIORAIN CO: Lot,160,050,073), unmodified human genomic DNA from unamplified control samples, and template control (containing all components of the reaction except template DNA).

Real-Time PCR software (ABI 7500 SDS v1.3.1) was used to analyze and collect the results. Using this software, the value of Cts was calculated for each sample (when the fluorescence level exceeded the threshold). The outcomes were obtained when the PCR efficiency was 85–100%, the correct slope value was -3 to 3.5, and R^2^ = 0.965–0.999.

The concentration of the methylated *Nrf2* and *ALU*-*C4* gene were calculated with standard curves. The normalized values for each sample were obtained by dividing the *Nrf2* gene values by the *ALU*-*C4* gene.

To calculate the percentage of methylation reference (PMR), the normalized values of each sample were divided by the normalized value of commercial human methylated DNA (positive control), and the result was reported as a percentage. [(*Nrf2/ALU-C4*) sample / (*Nrf2/ALU-C4*) positive control] ×100.

### Gene expression

This step included RNA extraction, complementary (cDNA) synthesis, primer design, and real-time PCR.

#### RNA extraction and cDNA synthesis

The placenta tissue RNAs were extracted with RNX-Plus solution. The RNA quality was tested by a Nano Drop spectrophotometer.

For the synthesis of cDNA according to the instruction of BonYakhte (BN-0011.37, Tehran, Iran), 1µL of RT enzyme, 1µL of dNTP, 4µL 5X RT buffer were mixed and added to DEPC-treated water to a volume of 20 µL. The product was mixed and incubated in a thermocycler for 10 min at 25 °C (pre-incubation step), 60 min at 42 °C (cDNA synthesis by reverse transcriptase enzyme), and 10 min at 70 °C (heat inactivation).

#### Primer design and real-time PCR

Specific primers were designed with Primer3 software based on the gene sequence. Real-time PCR amplification was carried out by Bonyakhte kit in a thermocycler containing SYBR™ Green PCR-Mastermix and ROX Dye solution.

The PCR reaction was performed in three steps according to the following temperature cycles:

- Pre-incubation, 95 ºC (3 min for genomic and 2 min for cDNA synthesis).
- 40 cycles as denaturation (95 ºC, 5 s) and annealing (50–60 ºC, 40 s).

The mean expression of *GAPDH*, a housekeeping gene, was used to normalize the input cDNA. The method for calculating the fold change was 2^−ΔΔCt^. The primers used are listed in Table 2.

**Table 2:** Primer sequences used for q-polymerase chain reaction

Primers	Sequence
*Nrf2* Forward Primer	5’-GCGACGGAAAGAGTATG-3’
*Nrf2* Reverse Primer	5’-GGGCAACCTGGGAGTA-3’
*GAPDH* Forward primer	5’-CTCTCTGCTCCTCCTGTTC-3’
*GAPDH* Reverse primer	5’-ACGACCAAATCCGTTGAC-3’

### Oxidative parameters assay

The total SOD enzyme activity in blood and supernatant of placenta tissue were evaluated which was measured in placenta tissue relative to total tissue protein. The level of placental protein was measured according to the instructions of the kit (Kiazist Co, KBCA-96, Tehran, Iran) and based on the BCA (Bicinchoninic Assay) method. The basis of this method is the protein’s ability to reduce Cu^+ 2^ to Cu^+ 1^ in an alkaline environment in the presence of Bicinchoninic acid, forming a Cu^+ 1^-BCA complex and producing a purple color that has absorbance at 560 nm.

The measurement of total antioxidant capacity [20] is based on the reduction of Cu^+ 2^ in the presence of antioxidants to Cu^+ 1^ and the formation of a colored product in the presence of a chromogen (readable at 450 nm).

Total oxidant status (TOS) examines the total oxidants, including free reactive oxygen species (ROS) or free nitrogen species, in a sample. This assay was performed according to the instructions of the KIAZIST kit (KTOS-96, KIAZIT, Tehran, Iran). The base of this assay is the oxidation of Fe^2+^ to Fe^3+^ in the presence of oxidants and the creation of a colored product by adding chromogen. This color is readable at a wavelength of 550–580 nm.

### Statistical analysis

Data were analyzed using SPSS software (version 16). To check the normal distribution of the data, the Kolmogorov-Smirnov test was used, and then the Mann-Whitney and Independent Sample T-tests were used to analyze the results. Pearson’s correlation coefficient was used to check the correlation between the results. The *P* value  < 0.05 was considered significant. To evaluate the sensitivity and the specificity of the *Nrf2* gene methylation level in the blood samples the receiver operating characteristic (ROC) was plotted.

## Results

### Demographic, clinical and biochemical results

In general, 140 blood samples (70 PE and 70 controls) and 40 placenta tissues (20 from PE group, and 20 from the control group) were taken from participants. The mean age of the patients was 30.7 ± 6.14 years old. There was a statistically significant difference between the two groups of control and PE (*P* > 0.001) in terms of gestational age and blood pressure (systolic and diastolic blood pressures) (Table 3).

**Table 3:** Demographic, clinical and biochemical characteristics of studied groups

Variable		Preeclampsiare women (*n* = 70) Mean ± SD	Healthy pregnant women (*n* = 70) Mean ± SD	*P* value*
Age (year)		29.56 ± 4.96	28.36 ± 6.37	0.113
Gestational age (week)		35.61 ± 3.56	38.5 ± 1.44	< 0.001
BMI (Kg/m^2^)		33.17 ± 5.68	29.93 ± 5.16	< 0.001
Systolic Blood Pressure (mmHg)		148.08 ± 17.00	112.85 ± 11.56	< 0.001
Diastolic Blood Pressure (mmHg)		91.66 ± 16.17	70.55 ± 10.52	< 0.001
Birth weight (g)		2622.5 ± 887.8	3390.7 ± 408	< 0.001
AST (IU/L)		28.2 ± 24.9	27.9 ± 12	0.96
ALT (IU/L)		24 ± 46.3	28.2 ± 26.9	0.8
ALP (IU/L)		338 ± 27	444.5 ± 150.8	0.1
Total Bilirubin (mg/dL)		0.62 ± 0.35	0.61 ± 0.17	0.96
Direct Bilirubin (mg/dL)		0.2 ± 0.07	0.19 ± 0.07	0.66
Proteinuria	+	44 (62.8%)	None	< 0.001
	++	15 (21.4%)	None	
	+++	9 (12.9%)	None	
	++++	2 (2.9%)	None	

AST: aspartate transaminase; ALT: alanine transaminase; ALP: alkaline phosphatase

* Independent sample t-test

### The PMR of the Nrf2 gene promoter region in blood samples and placenta tissues

The value of Cts of methylated DNA in PE and control groups was analyzed and calculated by Real-Time PCR software. The concentration of methylated DNA in the promoter region of the *Nrf2* gene (in blood and placenta samples) was calculated according to the PMR. The value of the PMR in the placenta tissues of the PE group (126.08 ± 15.54) was higher than that in the control group (75.12 ± 10.64) (*P* = 0.010). However, comparing blood samples indicated no significant difference between the PE group (87.12 ± 5.81) and control group (71.89 ± 5.11) in terms of PMR.

### Expression of the Nrf2 gene by 2^−∆∆CT^ method

The analysis of *Nrf2* gene expression data by the Relative Expression Software Tool (versions REST-2009) showed a significant decrease in the expression of this gene in the placenta tissues of preeclampsia women compared to the that in the control group (*P* = 0.014). However, comparing the expression of this gene in the blood samples of patients and controls was not statistically significant (Table 4).

**Table 4:** Expression of *Nrf2* gene in placenta tissue and blood samples of preeclampsia patients and controls

Gene	Sample	Fold change	*P*-value*
*Nrf2*	Placenta	0.124	0.014
*GAPDH*		1.000	
*Nrf2*	Blood	0.404	0.066
*GAPDH*		1.000	

* Independent sample t-test

### Correlation of Nrf2 gene expression and its methylation with the occurrence of preeclampsia

The results of the relationship between PMR and the expression of *Nrf*_*2*_ gene in placenta tissue samples of preeclamptic mothers and the control group showed that the relationship between the percentage of methylation and *Nrf*_*2*_ gene expression is significant. That is, with an increase in gene methylation, a decrease in gene expression. (*r* = 0.168).

### Sensitivity and specificity of the Nrf2 gene PMR index measurement

**Fig. 1:**
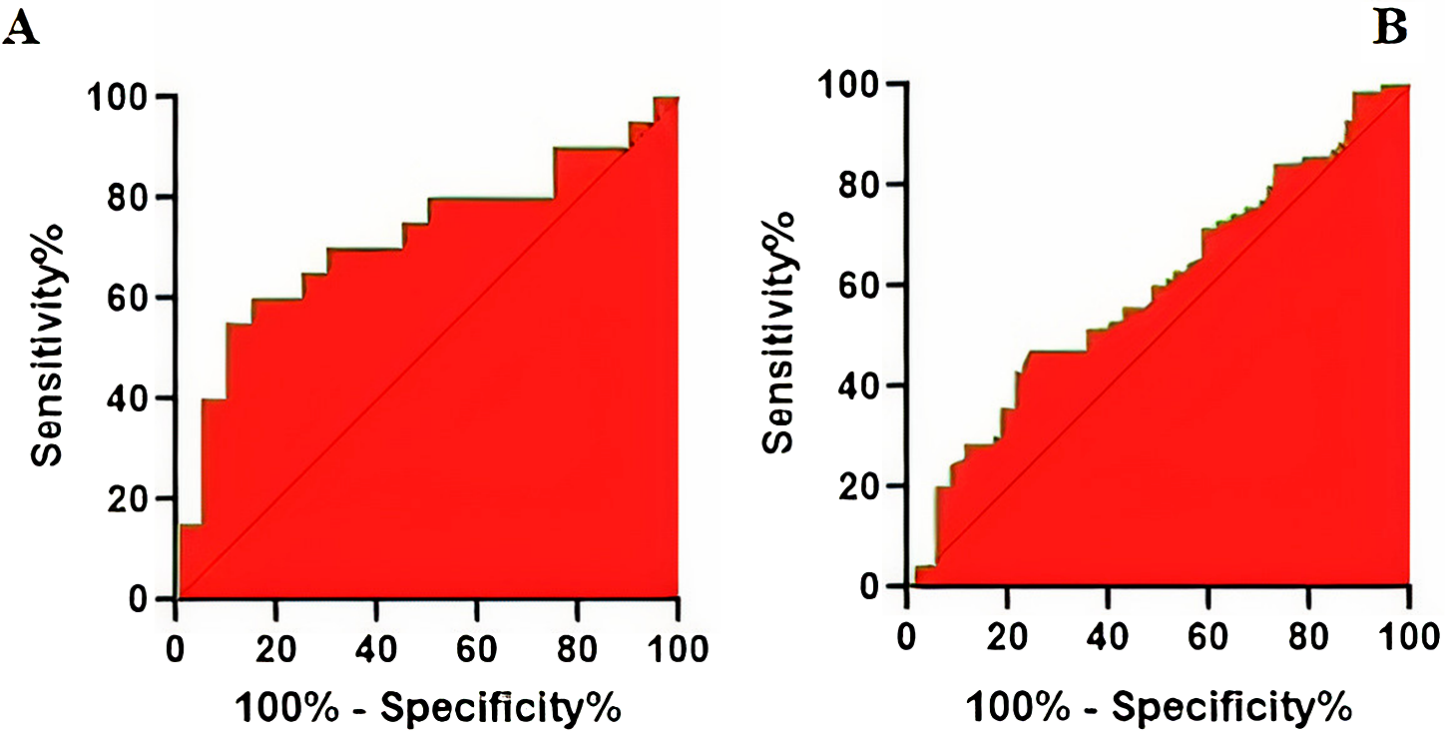
The specificity and sensitivity of PMR value of the *Nrf2* gene in the placenta tissue of preeclampsia patients using Graph Pad Prism v.9.0 software and analysis by ROC curve. The area under the curve is 0.72; the sensitivity is 75%; the specificity is 55% (**A**); The specificity and sensitivity of PMR value of the *Nrf2* gene in the blood of preeclampsia patients using Graph Pad Prism v.9.0 software and analysis by ROC curve. The area under the curve is 0.599; the sensitivity is 51%; the specificity is 62% (**B**)

The area under the curve (AUC) of the ROC curve revealed the efficiency of the *Nrf2* gene PMR measurement in blood and placenta samples. Due to the significant specificity and sensitivity in both placenta tissue (75%, 55%) and blood (51%, 62%) samples, this factor could be suitable for early diagnosis of PE, and probably, the investigation in a larger number of the population will help to evaluate its sensitivity and specificity better (Fig. 1).

### Oxidative parameters

There was no statistically significant difference in the level of TAC in the blood sample of the PE group (5.17 ± 1.75 nmol/mL) compared to the control group (5.47 ± 1.44 nmol/mL). While in the placenta tissue, a significant decrease was observed in the PE group (0.715 ± 0.07 nmol/mL) compared to the control group (1.5 ± 0.2 nmol/mL) (*P* = 0.001).

A statistically significant increase in the level of TOS in the PE group (47.17 ± 2.64 nmol/mL) compared to the control group (38.33 ± 1.9 nmol/mL) was observed in placenta tissue (*P* = 0.007). Also, there was a significant increase in the level of TOS in the PE group (2.47 ± 0.01 nmol/mL) compared to the control group (1.79 ± 0.2 nmol/mL) in blood samples (*P* = 0.017).

## Discussion

In a case-control study, we compared the percentage of *Nrf2* gene methylation and the expression level of this gene in placenta tissue and blood samples in PE and healthy pregnant women. The results of this study revealed that the *Nrf2* gene, a protective factor against redox-mediated damages, undergoes epigenetic changes of DNA hypermethylation in the placenta of PE. On the other hand, the decrease in the expression of this gene and the changes in the level of oxidative parameters (TAC, TOS) could be related to this epigenetic alteration.

The BMI and blood pressure (systolic and diastolic) of PE women were higher than that of controls, and the term pregnancy in PE women was shorter than that of controls. According to international guidelines, preeclampsia presents with high blood pressure (systolic > 140 mmHg or diastolic > 90 mmHg or both) that is accompanied by proteinuria or organ dysfunction after the 20th week of pregnancy [23]. Previous research showed that disorder in the remodeling of uterine spiral arteries causes placental ischemia, oxidative stress, and placental villus growth disorder [6]. In such conditions, which usually happen in the last trimester of pregnancy, there is the release of placental factors into the mother’s circulation, the imbalance of pro-angiogenic and anti-angiogenic factors (such as a decrease in placental growth factor (*PIGF*) and an increase in soluble fms-like tyrosine kinase-*1(sFlt*-*1) and* soluble endoglin (sEng)), and a maternal systemic endothelial disorder that leads to vascular damage and high blood pressure [3]. The impaired placental blood supply that leads to intrauterine growth restriction or placental abruption, results in preterm birth or stillbirth [24]. One of the risk factors for PE is an increase in the BMI of pregnant women. Previous studies have shown that obesity is associated with insulin resistance, dyslipidemia, chronic inflammation, and oxidative stress, all of which are also present in women with PE. A high BMI independently, or in interaction with other factors disrupting endothelial function, is a risk factor for PE [25, 26]. In a meta-analysis study that investigated weight gain and obesity as risk factors for preeclampsia, high BMI was introduced as an independent factor; their possible hypothesis was that maternal obesity has a direct adverse effect on implantation [27].

Oxygen plays a substantial role in placental damage as observed in PE. An insufficient oxygen supply to the trophoblasts of the placenta has been suggested as one of the causes of molecular events leading to the following clinical manifestations [28]. In the early stages of implantation, the gestational sac is located in a low-oxygen situation, which is helpful for trophoblast proliferation. Before the invasion, the proliferating trophoblasts anchor the blastocysts to the maternal tissues and close the tips of the spiral arteries in the decidua. Finally, plaques of the spiral trophoblastic artery are destroyed, and the inter-villous space is formed. This space allows the entry of maternal blood, increasing oxygen tension, enhancing oxidative stress, and promoting the differentiation of trophoblasts from the proliferative phenotype to the invasive phenotype. This new form of trophoblasts invades and remodels the spiral arteries [29]. It has been shown that the expression level of markers of cellular oxygen deficiency (i.e. hypoxia-inducible factors-1α and − 2α) increases in the placenta of PE [30]. At the molecular level, there is an imbalance of ROS-production and antioxidants level in the placenta of PE. According to Zhuang et al. study, the expression and the activity of ROS increase in the placenta of PE and inhibit the Wnt/beta-catenin signaling pathway that causes trophoblast invasion [31]. Huang et al. showed that oxidative stress promotes the transcription of anti-angiogenic factors such as sFlt1 [32].

Generally, placental antioxidant mechanisms are disturbed in pregnant women by a decrease in the expression of *SOD* and glutathione peroxidase [33].

We found that the *Nrf2* gene PMR in the placenta of PE women was higher than that in the control group, and the expression of the *Nrf2* gene in the placenta of PE women was lower than that in the control group. *Nrf2* is a transcription factor that coordinates the basal and stress-induced activity of a wide variety of cellular defense genes. *Nrf2* regulates transcriptional components of the antioxidant systems including thioredoxin, glutathione, NADH regeneration, and heme metabolism [10]. Previous studies showed that epigenetic processes, especially DNA methylation, affect the function and the process of a normal pregnancy [34]. Meanwhile, reports of hypo methylation of the promoter regions of tissue inhibitor of metalloproteinase 3, serine protease inhibitor 3, and hyper methylation of other promoter regions such as H19 maternally imprinted non-coding RNA have been reported [35]. Nezu et al. (2017) showed that *Nrf*_*2*_ knockdown in the mice model of PE improved the survival in mothers and fetal, improving the fetal growth restriction (FGR) and increasing the oxidative DNA damage. They found that the deficiency of *Nrf*_*2*_ in this animal model causes an increase in the mRNA expression of angiogenic chemokines, cytokines related to inflammation, and an increase in ROS [36]. Mundal et al. (2022) in cases of PE associated with intrauterine growth restriction showed that despite the increased level of oxidative stress, there was no change in the protein expression of *Nrf*_*2*_ and Keap1 of the decidua. However, in cases of PE with normal intrauterine growth, the increase in oxidative stress of the decidua was associated with a decrease in the expression of *Nrf*_*2*_ and an increase in the expression of Keap1 in areas of the uterus with high trophoblastic density. They concluded that decidua oxidative stress is modulated by trophoblastic cells [37]. Acar et al. (2014) reported that in PE women, the expression of *Nrf*_*2*_ in syncytiotrophoblasts of stromal cells of villous and vascular endothelium is significantly reduced [38]. However, Wruck et al. found that *Nrf*_*2*_ expression was exclusively expressed in villous cytotrophoblast cells and not in syncytiotrophoblast cells. On the other hand, its expression in the cytotrophoblastic nucleus of the PE female placenta is higher than that of the control group. They concluded that the expression of *Nrf*_*2*_ in cytotrophoblast cells indicates that these cells are involved in the expression of *Nrf*_*2*_-dependent antioxidant genes under oxidative stress [39].

According to the American College of Obstetrics and Gynecology [23], to predict the preeclampsia and prevent severe consequences of this disease, screening is limited to taking a medical history. The considered records are a combination of maternal factors, mean arterial pressure, uterine-artery pulsatility index and PIGF [23]. However, evaluating the specific markers, especially in the third trimester of pregnancy, is not without grace. The most notable biomarkers are sENG, sFlt1, and PIGF, which have high sensitivity and specificity for early detection and prognosis of preeclampsia [40]. The application of biomarkers for prognosis and early diagnosis has encouraged researchers to investigate various maternal and fetal markers. In a study conducted on 402 patients with preterm singleton pregnancy by Rana et al., it was shown that the sFlt1/PIGF ratio of more than 85 has a positive predictive value of 59% in all patients and a positive predictive value of 74% in patients < 34 weeks with severe symptoms of PE [41]. We indicated the epigenetic changes of the *Nrf2* gene during pregnancy. However, the low sensitivity and low specificity of measuring this factor showed that this test alone is infeasible in predicting pregnancy complication in pregnant women.

## Conclusion

In this case-control study, we found that the *Nrf2* gene PMR in the placenta of PE women was higher than that of the control group, and the expression of the *Nrf2* gene in the placenta tissue of PE women was lower than that of the control group. Also, the levels of TAC, and TOS in the placenta sample of PE women was lower and higher than the control, respectively. Despite the epigenetic changes of the DNA (hypermethylation of the *Nrf2* gene), the low sensitivity and specificity of the *Nrf2* gene PMR assay showed that the selection of this gene alone has not enough efficacy in the diagnosis and prognosis of preeclampsia.

## Data Availability

All data generated or analyzed during this study are included in this published article.
